# Supplementary material for: Modeling glioblastoma heterogeneity as a dynamic network of cell states
Source: Mol Syst Biol. 2021 Sep 16;17(9):e10105. doi: 10.15252/msb.202010105 (PMC8444284; doi:10.15252/msb.202010105)
Supplement: Supplementary file 6 — Source Data for Figure 5 [file MSB-17-e10105-s004.zip › Figure5A_sourcedata/GSEA_3017/nosigdb_stateA.GseaPreranked.1622795026485/FELDMAN_2020_S-G2.html]

Details for gene set FELDMAN\_2020\_S-G2[GSEA]

|  || Dataset | state53017 |
| Phenotype | NoPhenotypeAvailable |
| Upregulated in class | na\_pos |
| GeneSet | FELDMAN\_2020\_S-G2 |
| Enrichment Score (ES) | 0.27983108 |
| Normalized Enrichment Score (NES) | 2.3270004 |
| Nominal p-value | 0.0 |
| FDR q-value | 4.1206382E-4 |
| FWER p-Value | 0.009 |
Table: GSEA Results Summary

  

Fig 1: Enrichment plot: FELDMAN\_2020\_S-G2      
 Profile of the Running ES Score & Positions of GeneSet Members on the Rank Ordered List

  

| PROBE | GENE SYMBOL | GENE\_TITLE | RANK IN GENE LIST | RANK METRIC SCORE | RUNNING ES | CORE ENRICHMENT || 1 | CDK1 |  |  | 3 | 1.006 | 0.0126 | Yes |
| 2 | MKI67 |  |  | 4 | 0.926 | 0.0275 | Yes |
| 3 | UBE2C |  |  | 7 | 0.793 | 0.0379 | Yes |
| 4 | KIF20B |  |  | 10 | 0.749 | 0.0475 | Yes |
| 5 | CEP55 |  |  | 11 | 0.747 | 0.0595 | Yes |
| 6 | BUB3 |  |  | 12 | 0.740 | 0.0714 | Yes |
| 7 | KPNA2 |  |  | 20 | 0.667 | 0.0739 | Yes |
| 8 | HIST1H4C |  |  | 21 | 0.659 | 0.0845 | Yes |
| 9 | IGFBP3 |  |  | 26 | 0.641 | 0.0901 | Yes |
| 10 | ASPM |  |  | 27 | 0.639 | 0.1003 | Yes |
| 11 | DNAJC9 |  |  | 34 | 0.615 | 0.1031 | Yes |
| 12 | CKS2 |  |  | 40 | 0.587 | 0.1066 | Yes |
| 13 | NCAPG |  |  | 44 | 0.556 | 0.1120 | Yes |
| 14 | HJURP |  |  | 47 | 0.539 | 0.1183 | Yes |
| 15 | CKS1B |  |  | 50 | 0.530 | 0.1245 | Yes |
| 16 | DBF4 |  |  | 55 | 0.514 | 0.1280 | Yes |
| 17 | RPL22L1 |  |  | 56 | 0.512 | 0.1363 | Yes |
| 18 | SMC3 |  |  | 58 | 0.504 | 0.1432 | Yes |
| 19 | CENPE |  |  | 62 | 0.496 | 0.1476 | Yes |
| 20 | MIS18BP1 |  |  | 64 | 0.495 | 0.1544 | Yes |
| 21 | CCNA2 |  |  | 65 | 0.495 | 0.1623 | Yes |
| 22 | TPX2 |  |  | 66 | 0.488 | 0.1702 | Yes |
| 23 | MYBL2 |  |  | 69 | 0.486 | 0.1756 | Yes |
| 24 | HNRNPF |  |  | 70 | 0.485 | 0.1834 | Yes |
| 25 | HIST1H1B |  |  | 77 | 0.475 | 0.1840 | Yes |
| 26 | VRK1 |  |  | 82 | 0.470 | 0.1868 | Yes |
| 27 | UBE2T |  |  | 83 | 0.470 | 0.1943 | Yes |
| 28 | DUT |  |  | 87 | 0.466 | 0.1983 | Yes |
| 29 | TUBB4B |  |  | 93 | 0.462 | 0.1998 | Yes |
| 30 | CENPF |  |  | 94 | 0.461 | 0.2072 | Yes |
| 31 | TOP2A |  |  | 95 | 0.457 | 0.2146 | Yes |
| 32 | HIST1H1D |  |  | 98 | 0.456 | 0.2195 | Yes |
| 33 | RBM17 |  |  | 105 | 0.447 | 0.2196 | Yes |
| 34 | ZWINT |  |  | 106 | 0.447 | 0.2268 | Yes |
| 35 | AURKB |  |  | 108 | 0.446 | 0.2328 | Yes |
| 36 | KIF23 |  |  | 116 | 0.436 | 0.2315 | Yes |
| 37 | DEPDC1 |  |  | 119 | 0.432 | 0.2361 | Yes |
| 38 | MELK |  |  | 123 | 0.427 | 0.2394 | Yes |
| 39 | HIST2H2AC |  |  | 131 | 0.419 | 0.2379 | Yes |
| 40 | HELLS |  |  | 135 | 0.417 | 0.2411 | Yes |
| 41 | RTKN2 |  |  | 141 | 0.413 | 0.2418 | Yes |
| 42 | GTSE1 |  |  | 146 | 0.411 | 0.2437 | Yes |
| 43 | ANLN |  |  | 152 | 0.407 | 0.2443 | Yes |
| 44 | MAD2L1 |  |  | 153 | 0.406 | 0.2508 | Yes |
| 45 | KIFC1 |  |  | 160 | 0.402 | 0.2502 | Yes |
| 46 | ARHGAP11A |  |  | 162 | 0.399 | 0.2555 | Yes |
| 47 | MCM7 |  |  | 171 | 0.394 | 0.2524 | Yes |
| 48 | NDC80 |  |  | 173 | 0.393 | 0.2575 | Yes |
| 49 | BAMBI |  |  | 175 | 0.391 | 0.2626 | Yes |
| 50 | CKLF |  |  | 183 | 0.388 | 0.2606 | Yes |
| 51 | CMSS1 |  |  | 191 | 0.381 | 0.2584 | Yes |
| 52 | KIF22 |  |  | 197 | 0.377 | 0.2586 | Yes |
| 53 | CDCA3 |  |  | 203 | 0.372 | 0.2587 | Yes |
| 54 | PTMA |  |  | 206 | 0.371 | 0.2623 | Yes |
| 55 | TRIP13 |  |  | 209 | 0.370 | 0.2659 | Yes |
| 56 | SMC4 |  |  | 217 | 0.367 | 0.2635 | Yes |
| 57 | RAB13 |  |  | 220 | 0.366 | 0.2670 | Yes |
| 58 | SPC25 |  |  | 226 | 0.363 | 0.2669 | Yes |
| 59 | CENPA |  |  | 243 | 0.352 | 0.2537 | Yes |
| 60 | H2AFZ |  |  | 245 | 0.351 | 0.2582 | Yes |
| 61 | FEN1 |  |  | 259 | 0.343 | 0.2484 | Yes |
| 62 | TUBB |  |  | 264 | 0.340 | 0.2491 | Yes |
| 63 | SLC25A5 |  |  | 266 | 0.340 | 0.2534 | Yes |
| 64 | SNRPE |  |  | 271 | 0.338 | 0.2541 | Yes |
| 65 | PLP1 |  |  | 272 | 0.338 | 0.2595 | Yes |
| 66 | LRR1 |  |  | 273 | 0.337 | 0.2649 | Yes |
| 67 | CLSPN |  |  | 274 | 0.336 | 0.2703 | Yes |
| 68 | ESCO2 |  |  | 282 | 0.333 | 0.2674 | Yes |
| 69 | ORC6 |  |  | 283 | 0.333 | 0.2728 | Yes |
| 70 | CACYBP |  |  | 288 | 0.330 | 0.2734 | Yes |
| 71 | HSPD1 |  |  | 291 | 0.328 | 0.2763 | Yes |
| 72 | GGCT |  |  | 298 | 0.326 | 0.2744 | Yes |
| 73 | MND1 |  |  | 304 | 0.324 | 0.2737 | Yes |
| 74 | BRCA2 |  |  | 308 | 0.323 | 0.2754 | Yes |
| 75 | CENPN |  |  | 310 | 0.323 | 0.2794 | Yes |
| 76 | HNRNPDL |  |  | 315 | 0.320 | 0.2798 | Yes |
| 77 | PLAU |  |  | 347 | 0.310 | 0.2483 | No |
| 78 | PRC1 |  |  | 350 | 0.310 | 0.2509 | No |
| 79 | POLR3K |  |  | 351 | 0.309 | 0.2558 | No |
| 80 | ANP32B |  |  | 357 | 0.307 | 0.2549 | No |
| 81 | CDCA2 |  |  | 369 | 0.304 | 0.2468 | No |
| 82 | CKAP2 |  |  | 379 | 0.301 | 0.2410 | No |
| 83 | RPA3 |  |  | 381 | 0.300 | 0.2446 | No |
| 84 | RRM2 |  |  | 383 | 0.300 | 0.2483 | No |
| 85 | CENPW |  |  | 386 | 0.299 | 0.2507 | No |
| 86 | TEX30 |  |  | 388 | 0.298 | 0.2543 | No |
| 87 | HMGA1 |  |  | 397 | 0.295 | 0.2496 | No |
| 88 | PA2G4 |  |  | 402 | 0.293 | 0.2496 | No |
| 89 | HMGB1 |  |  | 403 | 0.292 | 0.2543 | No |
| 90 | HIST1H2AH |  |  | 412 | 0.290 | 0.2495 | No |
| 91 | HMGB2 |  |  | 424 | 0.288 | 0.2412 | No |
| 92 | POP7 |  |  | 427 | 0.287 | 0.2434 | No |
| 93 | NUSAP1 |  |  | 430 | 0.286 | 0.2457 | No |
| 94 | NUF2 |  |  | 434 | 0.285 | 0.2467 | No |
| 95 | MIS18A |  |  | 439 | 0.284 | 0.2466 | No |
| 96 | LMNB1 |  |  | 442 | 0.282 | 0.2487 | No |
| 97 | CDC45 |  |  | 446 | 0.282 | 0.2497 | No |
| 98 | FBL |  |  | 451 | 0.281 | 0.2495 | No |
| 99 | SMS |  |  | 463 | 0.278 | 0.2410 | No |
| 100 | HSPE1 |  |  | 465 | 0.277 | 0.2443 | No |
| 101 | ATAD2 |  |  | 470 | 0.275 | 0.2440 | No |
| 102 | SHCBP1 |  |  | 477 | 0.274 | 0.2413 | No |
| 103 | PGP |  |  | 503 | 0.270 | 0.2162 | No |
| 104 | KIF4A |  |  | 504 | 0.269 | 0.2205 | No |
| 105 | UBE2S |  |  | 507 | 0.269 | 0.2224 | No |
| 106 | PDCD5 |  |  | 526 | 0.265 | 0.2055 | No |
| 107 | SKA3 |  |  | 531 | 0.264 | 0.2050 | No |
| 108 | RANBP1 |  |  | 536 | 0.263 | 0.2045 | No |
| 109 | SRSF2 |  |  | 542 | 0.262 | 0.2028 | No |
| 110 | FBXO5 |  |  | 557 | 0.258 | 0.1905 | No |
| 111 | SPDL1 |  |  | 560 | 0.258 | 0.1923 | No |
| 112 | UBE2I |  |  | 564 | 0.257 | 0.1929 | No |
| 113 | SOX9 |  |  | 577 | 0.254 | 0.1828 | No |
| 114 | TMPO |  |  | 584 | 0.253 | 0.1798 | No |
| 115 | RNF19A |  |  | 591 | 0.252 | 0.1767 | No |
| 116 | RPS27L |  |  | 688 | -0.295 | 0.0683 | No |
| 117 | AKAP12 |  |  | 690 | -0.296 | 0.0718 | No |
| 118 | S100B |  |  | 696 | -0.299 | 0.0707 | No |
| 119 | MALAT1 |  |  | 702 | -0.302 | 0.0697 | No |
| 120 | SCD5 |  |  | 729 | -0.324 | 0.0442 | No |
| 121 | SPARC |  |  | 782 | -0.369 | -0.0112 | No |
| 122 | DCBLD2 |  |  | 791 | -0.375 | -0.0146 | No |
| 123 | BEX1 |  |  | 802 | -0.385 | -0.0202 | No |
| 124 | FTL |  |  | 825 | -0.409 | -0.0396 | No |
| 125 | SDCBP |  |  | 845 | -0.438 | -0.0549 | No |
| 126 | UPP1 |  |  | 867 | -0.467 | -0.0722 | No |
| 127 | NDFIP1 |  |  | 882 | -0.510 | -0.0805 | No |
| 128 | GPM6B |  |  | 897 | -0.542 | -0.0883 | No |
| 129 | SAT1 |  |  | 909 | -0.573 | -0.0921 | No |
| 130 | ENC1 |  |  | 922 | -0.621 | -0.0963 | No |
| 131 | TTYH1 |  |  | 932 | -0.652 | -0.0964 | No |
| 132 | CLU |  |  | 938 | -0.685 | -0.0913 | No |
| 133 | CDH2 |  |  | 947 | -0.762 | -0.0885 | No |
| 134 | IFITM3 |  |  | 954 | -0.830 | -0.0822 | No |
| 135 | LYPD1 |  |  | 955 | -0.839 | -0.0688 | No |
| 136 | CDKN1A |  |  | 969 | -0.987 | -0.0682 | No |
| 137 | FABP7 |  |  | 971 | -0.994 | -0.0535 | No |
| 138 | CTGF |  |  | 976 | -1.182 | -0.0392 | No |
| 139 | ID3 |  |  | 981 | -1.505 | -0.0197 | No |
| 140 | IGFBP5 |  |  | 983 | -1.595 | 0.0047 | No |
Table: GSEA details [plain text format]

  

Fig 2: FELDMAN\_2020\_S-G2: Random ES distribution      
 Gene set null distribution of ES for **FELDMAN\_2020\_S-G2**
